# Supplementary material for: mTORC1 activation in presumed classical monocytes: observed correlation with human size variation and neuropsychiatric disease
Source: Aging (Albany NY). 2024 Jul 26;16(14):11134–50. doi: 10.18632/aging.206033 (PMC11315394; doi:10.18632/aging.206033)
Supplement: Supplementary Figures [file aging-16-206033-s001.pdf]

## SUPPLEMENTARY FIGURES

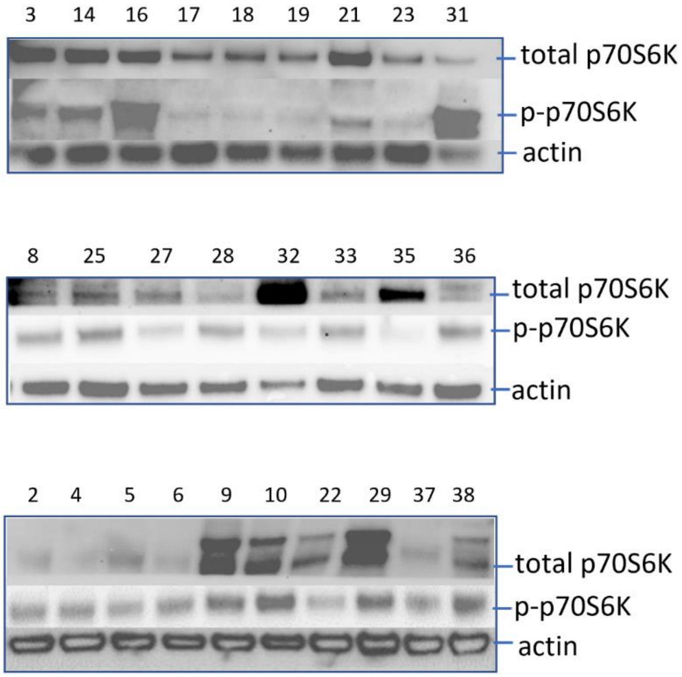


**Supplementary Figure 1. Western blots to assay phosphorylated p70S6K/total p70S6K ratio.** Western blot of p70S6K, phosphorylated p70S6K, and actin to assay the phosphorylated p70S6K/total p70S6K ratio. The actin quantity could not be estimated for the 10 samples in the bottom panel, limiting actin as control for aliquot sizing to 17 patients. Each number refers to a patient. p70S6K, p70 ribosomal S6 kinase; p-p70S6K, phosphorylated p70S6K.

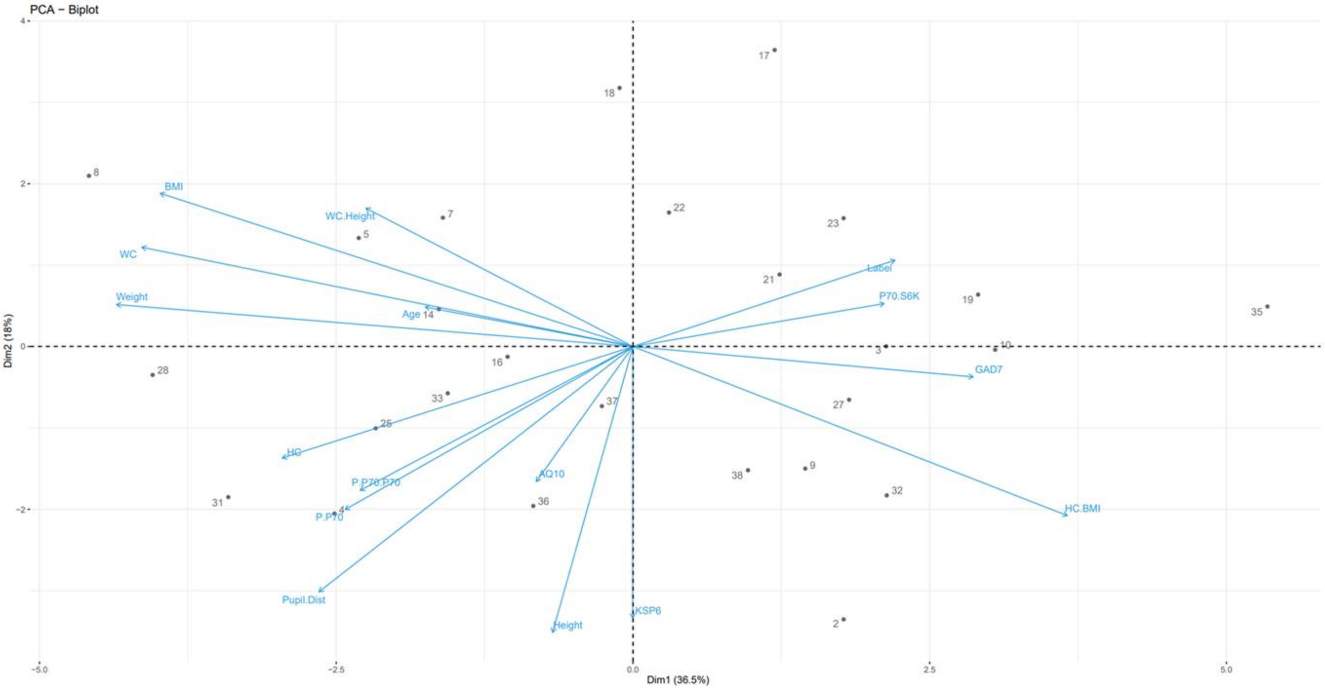


**Supplementary Figure 2. PCA analysis on the entire dataset.** Each dot represents a patient. Dimension 1 (Dim1) explains 36.5% of the variance and dimension 2 (Dim2) explains 18% of the variance. "Label" indicates the ketamine response. AQ10, 10-item Autism Spectrum Quotient; BMI, body mass index; GAD7, 7-item Generalized Anxiety Disorder; KSP6, HC, head circumference; HC BMI, ratio head circumference/BMI ratio; Karolinska Scales of Personality; PCA, principal component analysis; Pupil Dist, pupil distance; P.P70, phosphorylated p70S6K; P.P70.P70, ratio of phosphorylated p70S6K/total p70S6K; WC, waist circumference; WC Height, ratio waist circumference/height.
