# Supplementary material for: mTORC1 activation in presumed classical monocytes: observed correlation with human size variation and neuropsychiatric disease
Source: Aging (Albany NY). 2024 Jul 26;16(14):11134–50. doi: 10.18632/aging.206033 (PMC11315394; doi:10.18632/aging.206033)
Supplement: Supplementary Tables [file aging-16-206033-s002.pdf]

SUPPLEMENTARY TABLES

Supplementary Table 1. Clinical parameters and corresponding AUC values for ketamine response prediction.

Clinical parameter	AUC (95% CI)
Phosphorylated p70S6K	0.80 (0.53–0.97)
Ratio phosphorylated p70S6K/total p70S6K	0.78 (0.59–0.94)
Weight	0.76 (0.54–0.93)
Head circumference	0.74 (0.50–0.93)
Pupil Distance	0.74 (0.50–0.93)
Waist circumference	0.74 (0.48–0.93)
AQ-10 score	0.73 (0.51–0.91)
BMI	0.72 (0.52–0.91)
Ratio head circumference/BMI	0.72 (0.48–0.90)
GAD-7 score	0.67 (0.47–0.91)
Height	0.66 (0.48–0.91)
Ratio waist circumference/height	0.64 (0.45–0.91)
KSP-6 score	0.61 (0.45–0.90)
Age	0.58 (0.42–0.90)
p70S6K expression	0.51 (0.41–0.90)

AUC, area under the curve; AQ-10, 10-item Autism Spectrum Quotient; BMI, body mass index; CI, confidence interval; GAD-7, 7-item Generalized Anxiety Disorder; KSP-6, Karolinska Scales of Personality.

Supplementary Table 2. Ranking of clinical parameters based on random forest modelling.

Clinical parameter	Mean decrease accuracy
Phosphorylated p70S6K	0.034579
Height	0.011966
Ratio head circumference/BMI	0.010941
AQ-10 score	0.00569
BMI	0.003914
GAD-7 score	0.002192
Head circumference	0.001602
Weight	0.001418
Pupil distance	-0.00026
Ratio waist circumference/height	-0.00054
Age	-0.00058
Ratio Phosphorylated p70S6K/total p70S6K	-0.00087
p70S6K expression	-0.00336
KSP-6 score	-0.00511
Waist circumference	-0.00824
