# Supplementary material for: mTORC1 activation in presumed classical monocytes: observed correlation with human size variation and neuropsychiatric disease
Source: Aging (Albany NY). 2024 Jul 26;16(14):11134–50. doi: 10.18632/aging.206033 (PMC11315394; doi:10.18632/aging.206033)
Supplement: Supplementary Data [file aging-16-206033-s003.pdf]

SUPPLEMENTARY DATA

Informed consent form

RAPAMYCIN RIGHT-TO-TRY

RAPAMYCIN FOR PREVENTION AGE-RELATED PSYCHIATRIC DECLINE
RIGHT TO TRY IN WASHINGTON STATE CONSENT FORM

1/17/21

"Section 1: The legislature finds that the process for approval of investigational drugs, biological products, and devices in the United States protects future patients from premature, ineffective and unsafe medications and treatments over time, but the process often takes many years. Patients who have a terminal illness do not have the luxury of waiting until an investigational drug, biological product or device receives final approval from the Unites States food and drug administration. The legislature further finds that patients who have a terminal illness should be to pursue the preservation of their own lives…."

1. _____ I agree that I have a serious or immediately life threatening disease (reasonable likelihood of death within 6 months OR premature death is likely without treatment)
2. _____I understand that I have failed a reasonable subset of established treatments for my illness and/or unwilling to pursue high risk heroic treatments
3. _____I understand that there are no major therapeutic advances available in the pipeline in terms of local clinical trials.
4. ____I understand in the WORST CASE rapamycin could produce immunosuppression, damage to the lining of the mouth and stomach, worsen underlying diabetes, or fluid leak in the lungs or limbs. Many of these conditions could produce premature death. There may also be side effects of this drug that have not yet been discovered.
5. ___I understand in the BEST CASE rapamycin may slow the expected progression of my illness with age. There is additional animal and human data suggesting the potential for recovery of function across a wide variety of illnesses. Your individual probability of response cannot be estimated with current technology.
6. ___I understand that my insurance carrier may not be liable for treatment costs or any harm caused by rapamycin
